# Live magnetomyography and robotic-hand proxy control using a wearable triaxial optically pumped magnetometer

**DOI:** 10.1038/s41598-026-49442-x

**Published:** 2026-05-06

**Authors:** Rach Dawson, Sohaila Safavi, Marcin S. Mrozowski, Carolyn O’Dwyer, Dominic Hunter, Erling Riis, Paul. F. Griffin, Stuart Ingleby

**Affiliations:** 1 Department of Physics, SUPA, University of Strathclyde, Glasgow, G4 0NG United Kingdom; 2 Nuffield Department of Clinical Neurosciences, Oxford University Centre for Integrative Neuroimaging, FMRIB, Oxford, United Kingdom; 3 Fraunhofer Centre for Applied Photonics, Glasgow, United Kingdom

**Keywords:** OPM, Magnetomyography, Neural network, Human–machine interface, Biological techniques, Engineering, Neuroscience

## Abstract

Zero-field optically pumped magnetometers (OPMs) represent a promising modality in biomagnetism research due to their ultra-sensitive capabilities contained within compact sensor packages that enable contactless biomagnetic measurements. In this study, we utilized a zero-field OPM to simultaneously measure three-axis components of biomagnetic fields produced by muscle activity in the human forearm caused through independent finger movements, with the goal of recognizing multiple gestures using only a single magnetic sensor. We recorded muscle activity caused by the opening and closing of each finger 60 times, triggered by an auditory tone, and trained a neural network to classify the different finger movements. We utilized the triaxial OPM and trained neural network in a live-testing mode to replicate the participant’s hand gestures onto a robotic-hand proxy, with *≈ 1~*s delay, firstly for three fingers (little, middle, and thumb) and subsequently for all five fingers. Using a single OPM sensor, we captured, recognized, and replicated finger gestures from one participant in a controlled laboratory setting. In the three-gesture live test, all three finger movements were identified and replicated with ≥ 98% accuracy. In the five-gesture live test, four out of five fingers were identified and replicated with ≥ 90% accuracy, with the little finger being the most challenging to distinguish with a reduced 45% accuracy. These results demonstrate that a single zero-field OPM sensor in a triaxial measurement scheme can be effectively used for contactless recognition and replication of multiple finger movements, providing a proof-of-concept toward future wearable OPM-based magnetomyography systems.

## Introduction

Zero-field OPMs have gained credibility in the field of biomagnetism for their functional sensing capabilities^1–6^. With high magnetic sensitivity in the range of tens of femtotesla (10^−15^ T) and compact form factors (cm scale), zero-field OPMs are well-suited for a variety of biomagnetic measurements. Unlike other highly sensitive magnetometers, such as superconducting quantum interference devices (SQUIDs), OPMs do not require cryogenic cooling, making them far more practical for miniaturization and ultimately more suitable as a wearable technology^7–9^. Recent advances in OPM technology have demonstrated their effectiveness in measuring biomagnetic signals from the heart^10–14^, brain^15–19^, and muscles^20–22^, establishing them as a powerful tool for both diagnostic and research purposes. Beyond conventional diagnostic applications, OPMs also hold promise for studying functional muscle^20,22,23^ and nerve activity^24,25^

Electromyography (EMG) and magnetomyography (MMG) are techniques used to measure muscle activity, with EMG capturing electrical signals generated by muscles and MMG detecting the corresponding magnetic fields. EMG is a robust technique which is the current standard for non-invasive muscle measurement, but its poor spatial resolution^26^ often necessitates the use of dense sensor arrays^27^, increasing system complexity when resolving multiple gestures and extracting meaningful signals. MMG using OPMs offers advantages as magnetic fields, unlike electric potentials, are less distorted by the intervening tissue layers between the source and the sensor, and are not affected by electrode–skin interface noise^20,26,28,29^.

Magnetomyography (MMG) has increasingly been used as a complementary modality to EMG for assessing muscle activity, with studies showing that it can capture muscle activation patterns and task-dependent changes in muscle recruitment^30–32^. Recent developments in OPM technology have enabled highly sensitive MMG measurements without cryogenic requirements, facilitating closer proximity to biological sources and improved signal fidelity. Previous work has demonstrated MMG using multiple single-axis OPM sensors^20^, but such approaches typically require multiple sensors to obtain sufficient spatial information for gesture discrimination. Triaxial OPMs offer the opportunity to simplify measurement schemes and reduce hardware complexity while maintaining spatial resolution

Practical and wearable EMG-based Human–machine interfaces are demonstrated for applications such as prosthetic and robotic proxy control^33–36^ and virtual reality^37–40^. OPM-based MMG systems remain under-explored in these domains. The potential for reduced sensor count and system complexity, while maintaining or exceeding spatial and signal resolution, makes OPM MMG a compelling alternative. In this study, we utilize a zero-field OPM in a triaxial configuration to measure biomagnetic fields generated by muscle activity for multiple finger gesture recognition with a single sensor. This work aims to demonstrate the efficacy of this configuration for accurate and efficient finger gesture recognition, paving the way for advances in biomagnetic sensing and functional applications.

Here, we demonstrate a single-sensor MMG system using a triaxial OPM for multi-gesture classification and real-time robotic control. Unlike previous multi-sensor approaches, this work integrates triaxial magnetic field acquisition, convolutional neural-network based classification, and real-time robotic actuation within a single framework, enabling gesture decoding and prosthetic-style control from one compact sensor in a controlled setting.

**Fig. 1:**
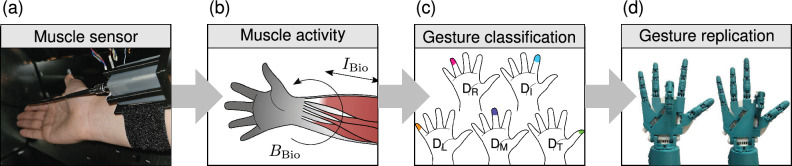
Experimental overview as indicated by four distinct steps. (**a**) A muscle sensor is fixed to the participant’s forearm in the desired location to record muscle activity. Pictured is a photograph of the OPM securely attached to a participant using a 3D-printed clip and Velcro, inside a five-layer magnetic shield. (**b**) The participant moves to a specific gesture resulting in muscle activity along the forearm inducing a bioelectric current, $I_\mathrm{{Bio}}$ and a corresponding biomagnetic field, $B_\mathrm{{Bio}}$, as indicated. (**c**) Through measurement of the muscle activity in real-time, the participants gesture (i.e the tensing and flexing of any finger as shown) is identified and classified using a machine learning model using a neural network. (**d**) Finally, the recognized gesture is sent to a robotic-hand proxy to replicate the gesture in real-time. Pictured is a photograph of the robot built for this purpose.

## Experimental methods

In this study, we aim to record and replicate real-time finger gestures of the right hand of a participant, and map these movements onto a robotic-hand proxy. The experimental scheme consists of four main phases, as seen in Fig. 1a muscle signal acquisition, using an appropriate measurement-dependent (EMG or MMG) muscle sensor secured to the participant’s forearm; (b) muscle activity induction, in which the participant performs a specific finger gesture that induces muscle activity along the forearm, generating bioelectric current, $I_\mathrm{{Bio}}$, and corresponding biomagnetic field, $B_\mathrm{{Bio}}$; (c) gesture classification, where the muscle activity is captured in real-time and classified into distinct categories using a neural network; and (d) gesture replication, in which the recognized gestures are transmitted to a robotic-hand proxy for real-time replication of the participant’s movements. Full description of each phase is contained in the following sections.

### Muscle sensors

We measure both the electrical and corresponding magnetic components of forearm muscle activity using an EMG sensor and an MMG sensor, respectively. The EMG sensor serves as a benchmark to validate the MMG signals captured by the OPM. This dual-sensor approach ensures the recorded signals originate from genuine muscle activity, establishing reliability in the biomagnetic measurements.

**Fig. 2:**
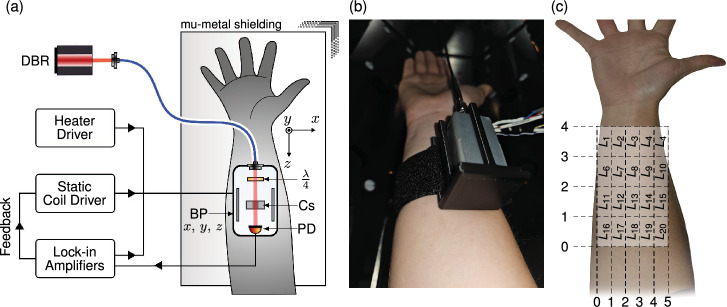
Overview of the OPM experimental setup. (**a**) The sensor head operates inside a five-layer mu-metal shield. Laser light from a Distributed Bragg Reflector (DBR) is delivered via an optical fibre to the sensor head. Bi-planar coils within the sensor provide three-axis static field control utilizing lock-in amplifiers for lock-in detection across all three axes. External electronics, including the Data Acquisition (DAQ) unit, coil driver^42^, lock-in amplifiers, and cell heater driver, are positioned outside the shield. (**b**) Photograph of the participant and the portable Cs magnetometer inside the magnetic shielding during measurements. (**c**) Photograph of the participant’s forearm with approximate sensor placement locations (L1 to L20) indicated.

We utilize a custom triaxial OPM^10,41^ to measure biomagnetic activity in the forearm during muscle activation. The setup of the OPM is shown in Fig. 2a, utilizing a single OPM in a triaxial operational mode to simultaneously obtain 3-axis magnetic field information. The sensor is housed in a five-layer mu-metal shield to attenuate ambient magnetic fields, achieving a low nT-level environment suitable for SERF-mode, near-zero-field operation. The internal residual magnetic field is nulled using bi-planar coils within the triaxial sensor head, which allow three-axis orthogonal field control. The sensor uses a micro-fabricated atomic vapor cell containing cesium atoms and 760 Torr of nitrogen buffer gas, heated to approximately 105 $^\circ$C. This setup operates in the spin-exchange relaxation-free (SERF) regime to minimize spin relaxation and enhance sensitivity. The atoms are pumped along the *z* axis using a 6 mW DBR laser tuned to the cesium D$_1$ resonance, with light elliptically polarized by a quarter-wave plate for optimal pumping. Light transmission through the vapor cell is measured by a photodiode, which is converted to magnetic field by scaling the transmission by the gradient in the axis-specific error signal of the absorptive feature. OPM operation in a closed-loop triaxial lock-in amplifier measurement scheme provides simultaneous measurement of $B_x$, $B_y$, and $B_z$ with sensitivities of 28 fT/$\sqrt{\text {Hz}}$, 35 fT/$\sqrt{\text {Hz}}$, and 97 fT/$\sqrt{\text {Hz}}$, respectively, over a 2 kHz bandwidth on each axis. For full sensor construction see^41^ and detailed description of the triaxial operation see^10^. The OPM is secured on the participant’s forearm using a custom 3D-printed clip and Velcro strap as seen in Fig. 2b, with a 1 mm air gap to minimize motion artifacts. The OPM is located on various points across the arm during signal classification, as shown in Fig. 2c, where the locations L1 to L20 are labelled; for all real-time measurements, the sensor is located at position L13.

#### EMG detector

A Seeeduino Grove EMG detector is used to measure electrical activity in the forearm during muscle activation. The EMG detector utilizes AMBU ECG wet gel electrodes placed on the participant’s forearm in the locations indicated in Fig. 2c, with a reference electrode on a neutral site. The analog output is recorded by an ADS1115 connected to an Arduino Nano Every, providing 16-bit resolution for measurements between 0-256 mV, sampled at 860 samples per second and averaged over every 10 samples. Signals were band-limited by the detector hardware and digitized via the ADS1115 analog-to-digital converter. These EMG recordings were used as a qualitative benchmark to confirm that the MMG signals reflected genuine muscle activity.

### Muscle activity

Muscle activity is characterized by the exchange of electrically charged particles, ions, within muscle fibres during contraction and relaxation. Surface EMG measures these electrical signals, while MMG detects the magnetic fields produced by these ionic currents^26^. MMG offers a contactless measurement method and is less affected by signal distortion due to tissue layers compared to EMG^28,29^. The typical frequency range for muscle activity is 20-200 Hz, with expected amplitudes in the picotesla to femtotesla range when measured by magnetometers^26,32,43,44^.

Signal duration depends on the type of movement, with fine motor tasks such as finger movements producing shorter, more distinct signals compared to larger muscle group activities. Muscle signals differ from nerve signals, which are more transient and serve as triggers for muscle activation. The distinction between nerve and muscle signals is essential for accurate interpretation of data. Nerve signals initiate muscle activity, while muscle signals represent ongoing contraction and force generation. Muscle contractions exhibit amplitude variations depending on the muscle and type of gesture^32^.

**Fig. 3:**
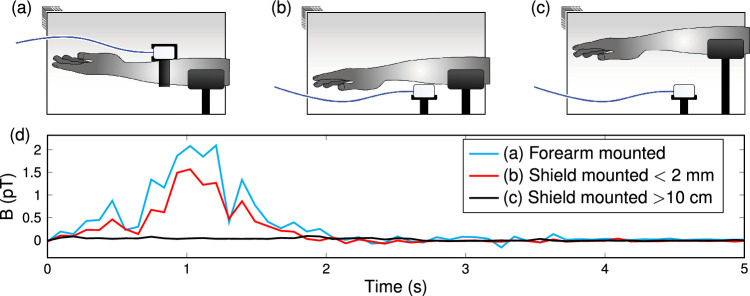
Comparison of measured biomagnetic signals under different sensor mounting conditions: (**a**) sensor directly mounted on participant’s forearm, (**b**) sensor rigidly fixed in the shield with the participant’s forearm within 2 mm, and (**c**) sensor fixed with the participant positioned over 10 cm away. (**d**) Averaged signal traces for D$_\mathrm{{M}}$ (middle finger flexion) recorded at sensor location **L13**, with each color representing a different mounting configuration.

#### Participant and setup

A right-handed, 25-year-old female participated in this study conducted in a controlled laboratory environment. The participant stood with her right arm relaxed inside a waist-height magnetic shield, supported on a 3D-printed mount positioned just above the elbow to minimize muscle tension. All data collection focused on individual finger movements of the right hand. Sessions were limited to batches of 20 gestures with rest intervals to prevent fatigue, and the OPM external sensor temperature was monitored to remain below 35*∘*C, ensuring participant comfort and safety throughout data collection. All methods were carried out in accordance with relevant institutional guidelines and regulations at the University of Strathclyde. The study involved a single participant, the lead researcher conducting the experiment. The work employed non-invasive, non-contact measurements and posed minimal risk to the participant. On this basis, the study was considered exempt from formal review by an institutional ethics committee. The participant provided fully informed written consent prior to participation.

**Fig. 4:**
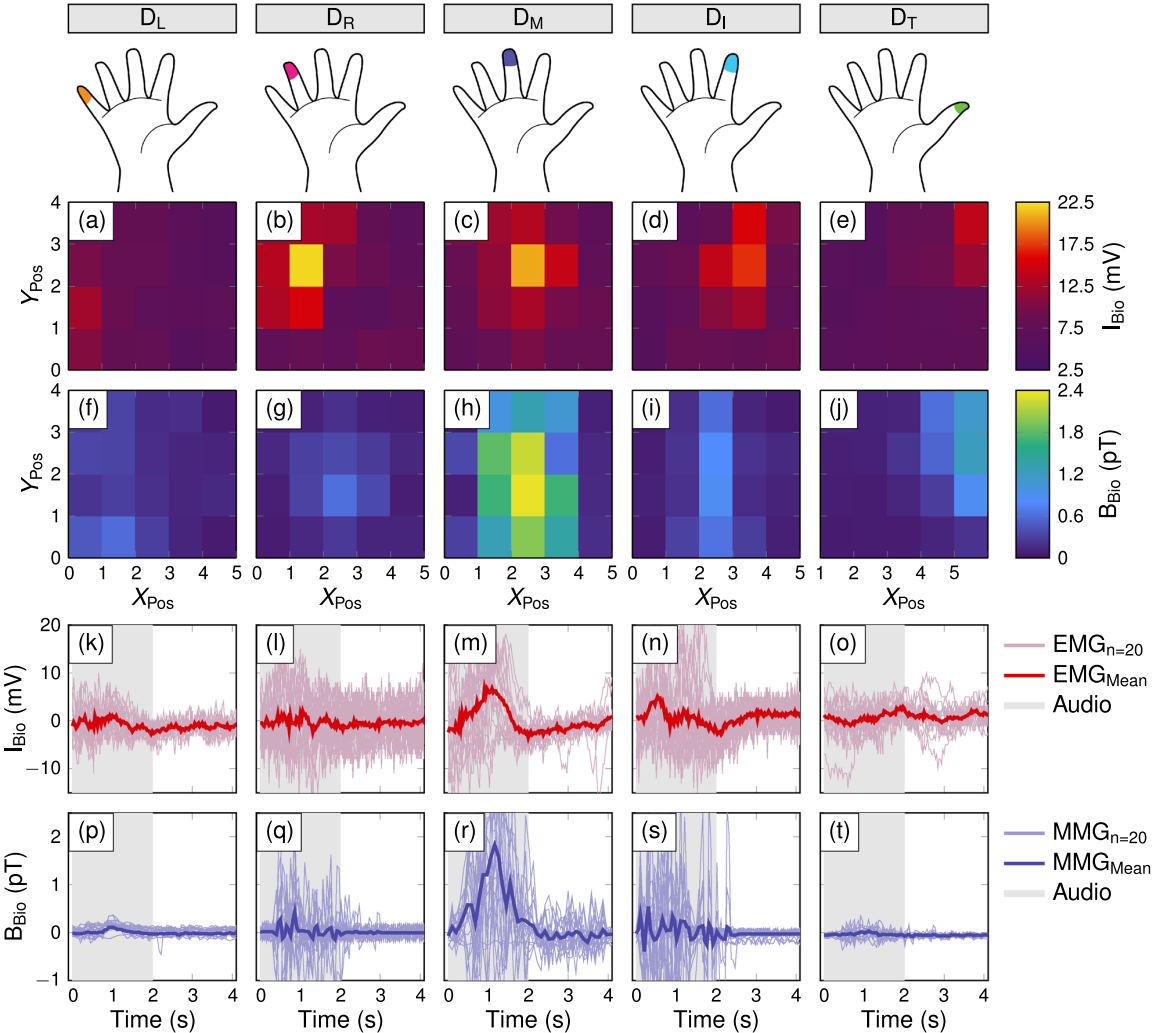
Forearm muscle activation mapping and time-resolved EMG/MMG signals across finger gestures. Each column corresponds to a distinct finger gesture: D$_\mathrm{{L}}$ (little finger) in (**a, f, k & p**), D$_\mathrm{{R}}$ (ring finger) in (**b, g, l & q**), D$_\mathrm{{M}}$ (middle finger) in (**c, h, m & r**), D$_\mathrm{{I}}$ (index finger) in (**d, i, n & s**), and D$_\mathrm{{T}}$ (thumb) in (**e, j, o & t**). Rows 1 & 2 display EMG and MMG heatmaps across 20 forearm locations (**L1** to **L20**) for each gesture, with colors representing signal intensity from maximum to minimum; lighter regions indicate stronger signals. Specifically, (a-e) represent EMG signal strength, while (**f-j**) display MMG signal strength across locations, mapping the areas of peak muscle activation for each gesture. Rows 3 and 4 illustrate time-varying EMG and MMG signals at the optimized location **L13**. (**k-o**) show 20 individual EMG traces (EMG$_\mathrm{{n=20}}$) for each gesture, overlaid with an average trace (EMG$_\mathrm{{Mean}}$). (**p**-**t**) similarly depict 20 MMG traces (MMG$_\mathrm{{n=20}}$) with the averaged signal (MMG$_\mathrm{{Mean}}$) highlighted. The 2-second auditory tone prompting the gesture is shaded in gray. This layout provides a comprehensive view of muscle activation distribution and time-resolved signal consistency for each targeted finger gesture.

#### Gestures

We selected five specific finger flexions to be performed; little D$_\mathrm{{L}}$, ring D$_\mathrm{{R}}$, middle D$_\mathrm{{M}}$, index D$_\mathrm{{I}}$, and thumb D$_\mathrm{{T}}$. Each gesture followed a 2-second sequence: a 1-second flexion (curling into the palm) and a 1-second relaxation back to an open hand. Movements were cued by a 2-second auditory tone every 5 seconds to ensure consistent timing. The participant was instructed to isolate the movement of the intended finger while keeping other digits relaxed. The forearm was supported to minimize motion and reduce unintended muscle activation. However, some physiological co-activation between neighboring fingers is expected. The sensor was positioned at an optimized location on the participant’s forearm based on preliminary testing, with data captured via NI-DAQ and processed in LabVIEW.

#### Signal validation

To validate the measured MMG signal, control tests were performed in various sensor mounting conditions to confirm we are measuring the muscle magnetic field, rather than any movement artifacts. We measured the flexion of the middle finger, D$_\mathrm{{M}}$, at location L13 for 20 repetitions in three mounting conditions. Firstly, with the sensor directly mounted on the participant’s forearm seen in Fig. 3a, secondly, with the sensor rigidly mounted to the shield, Fig. 3b, with the participant’s forearm within 2 mm (without direct contact). Lastly, we also measured with the sensor mounted to the shield, with the participant positioned over 10 cm away, Fig. 3b. The average of all 20 flexions time-varying signals is illustrated in Fig. 3d, in each mounting condition as indicated. Here, the average signal trace in the close-proximity shield mounted setup align with the forearm mounted configuration, confirmed by a low RMS error of $\approx ~12~\%$ between the two close-proximity measurements. However, the distant configuration produced no distinct measured signal (here with a high RMS error of $\approx ~80~\%$ between the forearm mounted measurement and the fixed > 10 cm distance measurement). Consistency across close-proximity mounting conditions, including the non-contact measurement, indicates the measured MMG signals originate from muscle activity rather than movement.

#### Optimal single-sensor placement

To identify the optimal placement for the single-sensor setup in live-gesture testing and validate the MMG measurements, we performed systematic measurements of EMG and MMG independently across a *5× 4* grid spanning 20 evenly spaced locations along the participant’s forearm, as seen in Fig. 2c. At each location we tested for all five finger flexions (D$_\mathrm{{L}}$, D$_\mathrm{{R}}$, D$_\mathrm{{M}}$, D$_\mathrm{{I}}$, & D$_\mathrm{{T}}$) for 20 repetitions, *n = 20*, per gesture. Muscle activation is triggered by a 2-second auditory tone, and the EMG and MMG sensors record the resulting time-varying signal.

From this scheme, forearm muscle activation heatmaps are generated, Fig. 4 rows 1 and 2, to visualize signal strength for each finger across all 20 locations, based on the average peak-to-peak amplitude extracted from the average trace of all 20 movements per gesture. The heatmaps for both EMG, Fig. 4 (row 1), and MMG, Fig. 4 (row 2), reveal comparable patterns of high signal intensity across locations, validating the MMG sensor’s capability to measure the same muscle activity as the EMG sensor.

We select L13 to provide an optimal pragmatic single-sensor placement for multi-gesture recognition in live-gesture testing, informed by the MMG heatmaps trends in Fig. 4 (row 2). Location L13 shows consistent measurable amplitudes across all five fingers, with relatively balanced signal strengths for gestures, whilst the little finger, Fig. 4f, and thumb, Fig. 4j, exhibited slightly lower signals compared to the others.

The time-varying EMG and MMG signals recorded at the optimized sensor location L13 are presented in Fig. 4 (rows 3 and 4). Fig. 4 k-o display 20 individual EMG traces, EMG$_\mathrm{{n=20}}$, for each gesture, along with the corresponding average trace, EMG$_\mathrm{{Mean}}$. Similarly, Fig. 4p-t illustrate 20 MMG traces, MMG$_\mathrm{{n=20}}$, with their averaged trace, MMG$_\mathrm{{Mean}}$. The time-domain traces for EMG and MMG at L13, Fig. 4 (rows 3 and 4) further validated this location choice, showing alignment in trends and waveform consistency across sensors.

**Fig. 5:**
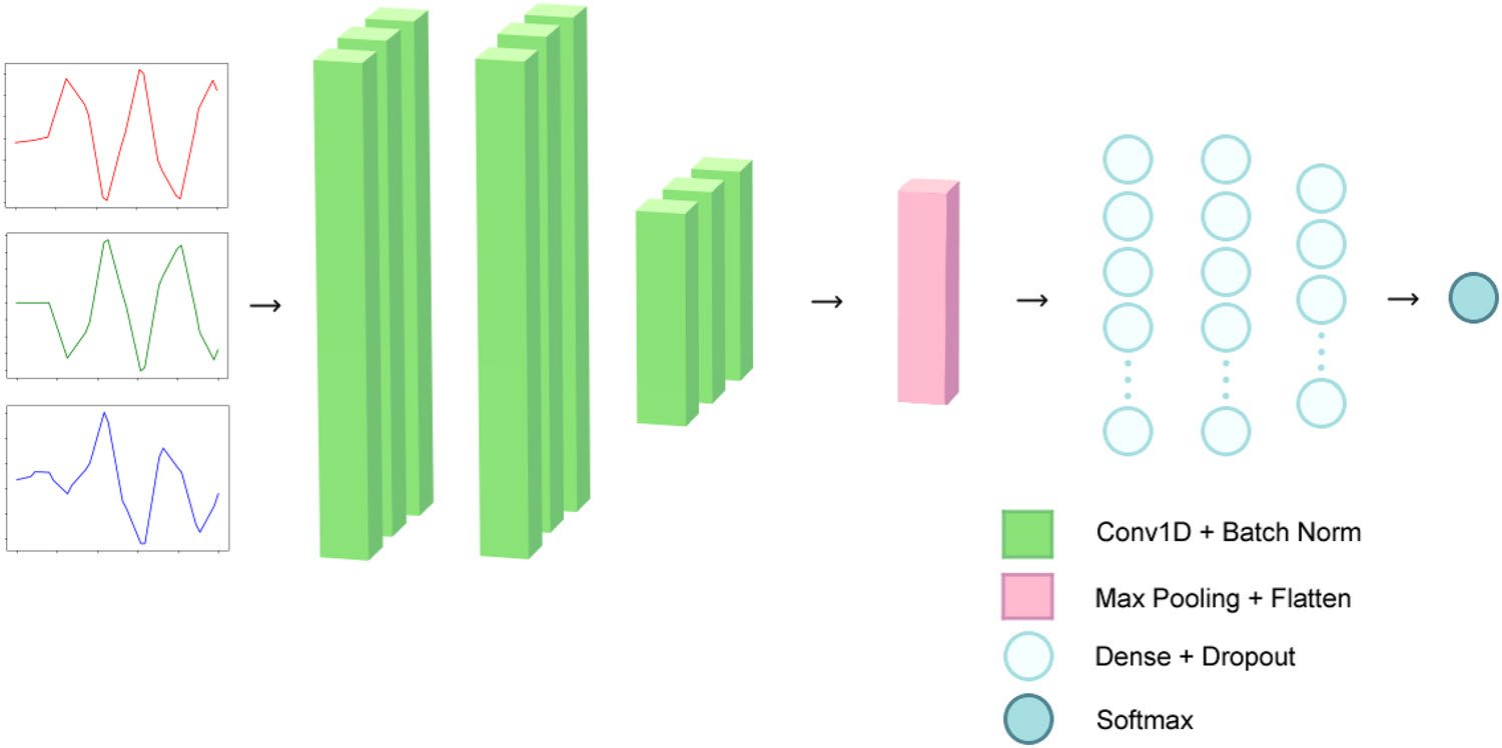
Convolutional neural network architecture. Input is passed through 3 Conv1D layers with 64, 64 and 16 filters respectively with each followed by Batch Normalization, then through max pooling + flattening, then goes through 3 Dense layers with 256, 256 and 128 filters each followed by Dropout layer with 0.5 dropout rate, and finally passed to Softmax layer for gesture classification.

The alignment of the EMG and MMG measurements in both the spatial heatmaps and time-domain signals supports the reliability of MMG as a non-contact biomagnetic measurement for real-time gesture classification (Fig. 5).

### Gesture classification

#### Neural network

We implemented a simple sequential 1D convolutional neural network with the AI library TensorFlow in a Python virtual environment. Using a training data set of 60 signals per gesture in a shuffled order, tensors of size $N \times T \times A$ (with N, the batch size of 15, T, the number of time steps in each signal and A, the number of axes) were passed into the network. The network, Figure 5 consisted of two 1D convolutional layers with 64 filters and a third with 16 filters, each with kernel size 3x3, rectified linear unit (ReLU) activation function and each followed by a Batch Normalization layer. This output was then passed to a 1D Max Pooling layer, flattened and passed to three Fully Connected (FC) layers with 256, 256 and 128 units, each using the ReLU activation function, L2 kernel reguliser and each followed by a Dropout layer with dropout rate 0.5. This was then passed to a final FC layer with *G* units representing the *G* possible outputs and softmax activation to categorize the output data. The model was then trained using the categorical cross-entropy loss function, Adam optimizer (with a learning rate of 0.0001, $\beta _1$ = 0.9 and $\beta _2$ = 0.999) and was trained over 80 epochs. To evaluate the model, validation data was used to compute its accuracy in predicting the recorded gestures with a training:validation ratio of 9:1. The same neural network architecture was used for both the three-gesture and five-gesture classification.

### Gesture replication

This section details the process of real-time gesture replication, in which classified gestures are replicated onto a robotic-hand proxy. After recognizing each gesture performed by the participant, the neural network transmits commands to the robotic system to execute the corresponding finger movements.

#### Robotic-hand proxy

The robotic-hand chosen for replication is based on the open-source InMoov project^45^, constructed using 3D-printed PLA parts, where each finger is hinged at the joints with nylon screws and controlled via high-tension fishing wire and driven by individual servo motors (HobbyKing Coreless Digital HV/MG/BB Servo). Each servo motor is calibrated to produce precise flexion and extension for each finger. Control is managed by an Arduino Uno Rev 3, interfacing with the neural network through serial communication, which receives classification commands from the neural network to actuate the designated finger.

#### Live-gesture experimental design

The live-gesture replication experiment is conducted in two tests, each consisting of 200 randomized gestures. Test A involves three specific gestures (D$_\mathrm{{L}}$, D$_\mathrm{{M}}$, and D$_\mathrm{{T}}$), while Test B includes all five gestures. A gesture is randomly selected by a LabVIEW script and audibly relayed to the participant. The participant performs the gesture in response to a 2-second auditory tone, with a 3-second interval between each movement for recovery. Each session was divided into 20-gesture batches with 5-minute breaks between batches to prevent fatigue.

The first 1-second of recorded triaxial magnetic data from the OPM is processed by the neural net and the recognized gesture is translated to the robotic-hand proxy, resulting in a *≃* 1 s delay between the participant movement initiating and the robotic-hand proxy replicating the movement. Performance is evaluated by comparing the neural network’s classified output with the target gesture; matching classifications are recorded as successful replications.

**Fig. 6:**
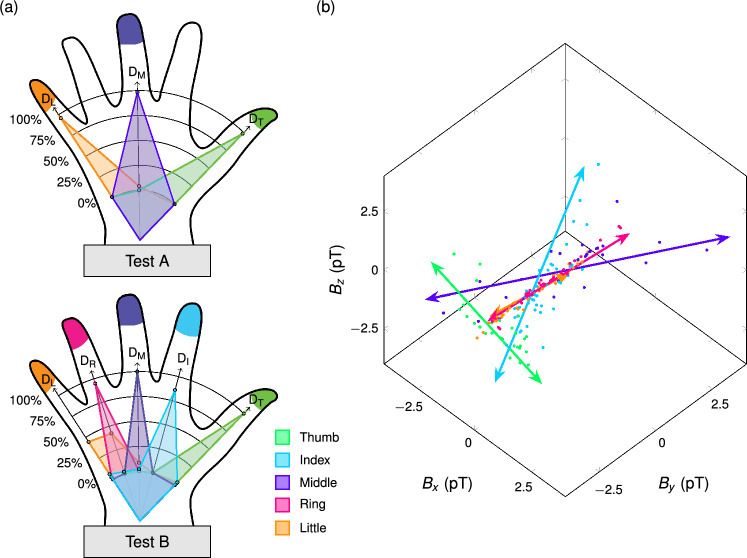
(**a**) Radar plot showing the correspondence between the participant’s intended finger movements (axes labelled with D$_\mathrm{{L}}$, D$_\mathrm{{M}}$, D$_\mathrm{{T}}$ for Test A and all five gestures for Test B) and the gestures recognized by the Neural Network. Axes are overlaid with directional labels corresponding to the finger’s position on the hand for clarity. Points on the radar plot indicate the percentage of correctly recovered gestures per finger, with the shaded areas highlighting the alignment between intended and recovered gestures. (**b**) Visualization of the recovered triaxial magnetic fields (B${_{x}}$, B${_{y}}$, B${_{z}}$) associated with each finger gesture, plotted in a 3D magnetic field landscape, where color represents the gesture type, arrows indicate the changing triaxial vector across the first 1 s of movement.

## Live-gesture identification and replication testing

We evaluated live-gesture recognition through two tests, A & B, targeting the accuracy of a neural network in identifying individual finger gestures live. Test A involved three gestures, D$_\mathrm{{L}}$, D$_\mathrm{{M}}$, and D$_\mathrm{{T}}$, while test B expanded to five gestures, including D$_\mathrm{{R}}$ and D$_\mathrm{{I}}$. In each test, classification accuracy was assessed by comparing the neural network’s output with the randomly selected intended gesture, as relayed before the auditory cue. Success is defined as alignment between the predicted and intended gestures, providing insight into the system’s precision across different gesture complexities.

Test A yielded high accuracy for all gestures, with each finger gesture (D$_\mathrm{{L}}$, D$_\mathrm{{M}}$, and D$_\mathrm{{T}}$) achieving recognition rates of *\ge* 98 

In test B, recognition accuracy remained high, > 90%, for D$_\mathrm{{R}}$, D$_\mathrm{{M}}$, D$_\mathrm{{I}}$, and D$_\mathrm{{T}}$, but dropped to 45% for D$_\mathrm{{L}}$, seen in Table 1). As seen in Fig. 6a: Test A, confusion between D$_\mathrm{{L}}$ and D$_\mathrm{{R}}$ accounted for the majority of errors, suggesting potential overlap in the neural network’s magnetic response signatures for these fingers. Despite this reduction for D$_\mathrm{{L}}$, the network maintained a consistent performance for other gestures, achieving robust classification in the five-gesture test configuration.

Figure 6b provides a 3D visualisation of the triaxial magnetic field responses ($B_x$, $B_y$, $B_z$) for each gesture, averaged across the first 1-second interval for 20 repetitions per gesture. A linear-fit across each average gesture provides an approximate triaxial vector across the first 1 s of movement, as indicated by arrow in Fig. 6b. This visualisation reveals that most gestures occupy distinct areas within the triaxial magnetic field space, supporting the system’s ability to discern individual finger movements. However, overlap is observed between D$_\mathrm{{L}}$ and D$_\mathrm{{R}}$, likely contributing to the reduced recognition accuracy for D$_\mathrm{{L}}$ in Test B. These findings confirm that the network can accurately recognise finger gestures in real-time, demonstrating the feasibility of neural network-based gesture recognition for robotic replication.

**Table 1:** Live-gesture recognition results for two distinct tests (A and B), as indicated.

Test	Gestures	*n*	D$_\mathrm{{L}}$ (%)	D$_\mathrm{{R}}$ (%)	D$_\mathrm{{M}}$ (%)	D$_\mathrm{{I}}$ (%)	D_T_ (%)
A	3	200	98		99		98
B	5	200	45	95	100	91	100

The number of gestures tested, gestures, and the total number of gestures recorded, *n*, during each test is defined. The gestures recorded are the flexion of the individual digits D$_\mathrm{{L}}$, D$_\mathrm{{R}}$, D$_\mathrm{{M}}$, D$_\mathrm{{I}}$, and D$_\mathrm{{T}}$ (little, ring, middle and index fingers, and the thumb respectively. The accuracy of gesture recognition of each gesture is indicated, found as a percentage of correctly recognized and replicated gesture by the robot, with respect to the total amount of times the participant created each gesture. Values in gray indicate gestures that were not measured during testing.

## Discussion

This study demonstrates the application of a single triaxial optically pumped magnetometer for real-time detection, classification, and robotic replication of individual finger gestures. Using a zero-field OPM in triaxial mode, we captured biomagnetic signals generated by specific finger movements, classified these gestures with a neural network, and relayed the output to a robotic-hand. The system achieved over 98% accuracy for a three-gesture test (D$_\mathrm{{L}}$, D$_\mathrm{{M}}$, and D$_\mathrm{{T}}$) and over 90% accuracy for four out of five finger gestures in the five-gesture test (Test B), with reduced accuracy for the little finger (D$_\mathrm{{L}}$).

The reduced accuracy for D$_\mathrm{{L}}$ likely arises from shared neuromuscular pathways between the little and ring fingers, both innervated by the ulnar nerve. This overlap contributed to similar biomagnetic response profiles, increasing misclassification between D$_\mathrm{{L}}$ and D$_\mathrm{{R}}$. Expanding the training dataset or adjusting the network architecture may help to further separate these signals. Additionally, the training data and live-gesture trials were recorded on different days, which may have introduced misalignment in sensor positioning. Implementing a more rigid sensor placement method or incorporating an in-situ calibration process before live testing could address this issue.

Using a single sensor for gesture recognition reduces system complexity and power requirements, making this approach promising for applications requiring a compact wearable setup. Potential applications include virtual reality interfaces^35,38^, robotic control and prosthetics^39,40^. Limitations of our approach include the need for a zero-field environment and optical fibre, which may be addressed by emerging unshielded OPM systems that can operate in the Earth’s magnetic field^46,47^ and use of compact in-sensor laser systems. Additionally, optimizing the system’s 1-second latency by reducing the data window required for accurate classification could in theory be tuned to decrease latency to a few hundred milliseconds, enabling faster response times.

This study represents a single-participant proof-of-concept conducted in a controlled magnetically shielded environment. The results do not establish cross-subject generalizability, as inter-individual variability in anatomy and muscle activation patterns may significantly affect signal characteristics. The sensitivity of biomagnetic measurements to sensor placement means that millimetre-scale shifts may affect classification performance. In practice, however, the model can be retrained for a given sensor placement in a short (several minute) calibration procedure (including data collection and training), which partially mitigates this constraint for wearable use.

We observed repeatability across trials within a session, but did not test the system across multiple sessions or over longer timescales. The requirement for magnetic shielding also limits immediate real-world applicability. Triaxial OPM schemes demonstrated in shielded environments^10^ may eventually support operation in less stringently shielded or even unshielded settings, provided that suitable active field control and noise-rejection strategies are developed.

This intrinsic overlap between little and ring finger activation limits how well a single sensor can separate these movements and may be mitigated by more selective feature extraction or by combining magnetomyography with additional sensing

This work demonstrates the feasibility in a controlled setting of using a single triaxial OPM system for real-time biomagnetic gesture recognition, with potential for broader applications in gesture-based control.

## Data Availability

The experimental data generated in this study, including the datasets used to generate the figures and to train the neural network models, All data created during this research is openly available from Pure Data. https://doi.org/10.15129/zenodo.18272094.
